# Rapid and reliable computational markers of decision-making for predicting daily smoking behavior and smoking cessation treatment outcomes

**DOI:** 10.1038/s41386-026-02448-5

**Published:** 2026-05-29

**Authors:** Jeung-Hyun Lee, Sang Ho Lee, Jaeyeong Yang, Hyeonjin Kim, Mark A. Pitt, Hyung Jun Park, Hee-Kyung Joh, Anna B. Konova, Woo-Young Ahn

**Affiliations:** 1 Department of Psychology, Seoul National University, Seoul, Republic of Korea; 2 School of Digital Humanities and Computational Social Sciences, KAIST, Daejeon, Republic of Korea; 3 Department of Psychology, The Ohio State University, Columbus, OH USA; 4 Center for Health Promotion, Samsung Medical Center, Seoul, Republic of Korea; 5 Department of Medicine, Seoul National University College of Medicine, Seoul, Republic of Korea; 6 Department of Family Medicine, Seoul National University Health Service Center, Seoul, Republic of Korea; 7 Department of Family Medicine, Seoul National University Hospital, Seoul, Republic of Korea; 8 Department of Psychiatry and the Brain Health Institute, Rutgers University, New Brunswick, NJ USA; 9 Department of Brain and Cognitive Sciences, Seoul National University, Seoul, Republic of Korea; 10 AI Institute, Seoul National University, Seoul, Republic of Korea

**Keywords:** Human behaviour, Predictive markers

## Abstract

Addiction involves rapidly fluctuating affective and value-based decision-making processes that undermine cessation efforts, yet capturing these dynamics at clinically meaningful timescales remains challenging. Standard cognitive decision-making tasks are time-intensive and require many trials to achieve reliable parameter estimates, limiting their use longitudinally and in real-world settings. Here, we developed a rapid, smartphone-based framework that integrates ecological momentary assessment (EMA) with adaptive design optimization (ADO), a Bayesian method that enables reliable estimation of computational decision-making parameters from as few as 20-30 trials per task. We tested this framework in *N* = 79 individuals undergoing a 5-6-week smoking cessation program, who completed daily ADO-based delay discounting and risk/ambiguity tasks alongside EMA surveys assessing smoking behavior, craving, stress, mood, anxiety, and medication adherence. At the day-to-day level, elevated craving, depressive symptoms, and ambiguity tolerance predicted increased smoking the following day, whereas lower discounting rates and reduced craving and stress predicted cessation success at treatment completion. For the latter, models using data from the first week achieved meaningful predictive performance (mean AUC = 0.76), approaching the upper-bound performance observed in models incorporating both task and survey data from the full study period (mean AUC = 0.83). Together, these findings demonstrate that rapid, low-burden, ADO-based delivery of decision-making tasks via EMA can capture clinically relevant, dynamic vulnerability states during smoking cessation treatment. This methodology offers a promising approach for identifying cognitive markers that may facilitate or inhibit cessation success and informing personalized, time-sensitive intervention strategies for nicotine addiction and related psychiatric disorders.

## Introduction

Smoking remains a major cause of morbidity and mortality worldwide [1]. Despite availability of various smoking cessation treatments, over two-thirds of smokers relapse within a year of a quit attempt [2, 3]. This high relapse rate emphasizes the need for identifying reliable predictors of successful cessation, particularly ones that smokers can integrate into their daily lives.

Previous research has identified demographic, motivational [4], and environmental factors [5], as well as stress, negative affect [6, 7], and nicotine use severity [8] and craving [9] as barriers to maintaining abstinence. More recently, there is growing interest in cognitive “computational markers” that predict clinical outcomes. These markers, which are parameters derived from fitting mathematical models to cognitive tasks like risk and delay discounting [10, 11], have proven useful in understanding neurocognitive mechanisms of a range of psychiatric conditions [10, 12, 13]. Such markers can enhance both clinical prediction and mechanistic insight into substance use disorders [14, 15], including smoking cessation [16, 17]. However, much of this research remains cross-sectional, limiting the ability to capture the dynamic and highly individualized nature of addiction recovery.

To address this gap, ecological momentary assessment (EMA) methods have been increasingly used to capture dynamic, within-person variations in psychological states (i.e., mood, craving, and stress) in naturalistic settings [6, 18]. EMA has already shown clinical utility in addiction research [19, 20], particularly for modeling temporal variations in addiction relevant self-reported psychological states [21, 22]. Yet, capturing similar dynamics with task-based cognitive markers remains a significant challenge.

Progress in this area has been limited by at least two key factors: First, traditional decision-making tasks are often lengthy and repetitive, imposing significant demands on participant motivation that can reduce data quality, particularly in clinical populations [23]. This calls for alternative methods that retain the rigor of laboratory-based measures while being feasible for shorter and more frequent deployment in real-world settings. Second, to our knowledge, no previous study has successfully combined self-reported surveys and computational markers on a daily and longitudinal basis to predict smoking behavior. Such densely sampled data could capture finer temporal dynamics in smoking cessation or relapse, providing unique and clinically actionable insights into the cognitive processes underlying addiction, as suggested in other substances [20]. Identifying critical moments and individual-specific decision patterns can guide real-time, personalized interventions tailored to individual trajectories, enhancing treatment effectiveness and long-term cessation success.

**Fig. 1:**
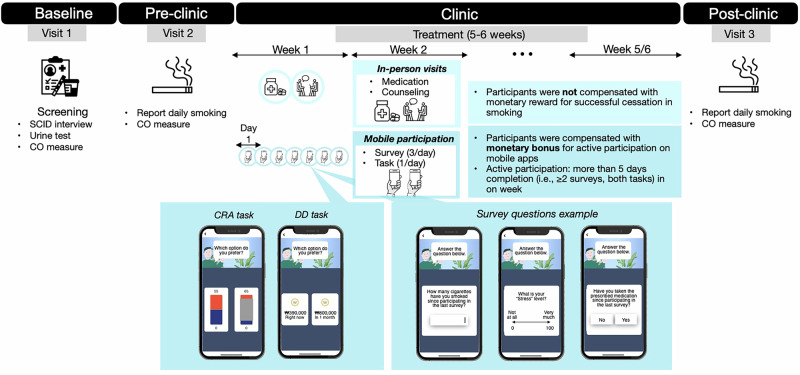
Study procedures. Participants who passed the screening procedures during an initial lab visit made an additional two visits to the lab. During treatment, participants attended weekly in-person medication prescribing and counseling sessions at a university health center and participated in daily EMA surveys and behavioral tasks. Before and again at the end of the treatment period (pre- and post-clinic), participants visited the lab and provided objective measures of exhaled carbon monoxide and reports on daily smoking behavior. On the bottom panel are sample screenshots of daily tasks and survey questions: CRA task (Choice under risk and ambiguity), DD task (Delay discounting).

To overcome these limitations, we conducted a longitudinal study in which individuals undergoing smoking cessation completed daily decision-making tasks alongside EMA surveys of psychological states (Fig. 1). We adapted two well-established tasks previously linked to drug reuse in laboratory-based studies: the delay discounting task [24], which measures temporal delay discounting and choice impulsivity [25, 26], and the choice under risk and ambiguity (CRA) task [27], which assesses risk and ambiguity tolerance [14]. We used the adaptive design optimization (ADO [28]) methodology—a machine-learning algorithm based on a Bayesian framework that selects the most informative task stimulus combinations in real-time—to shorten the tasks’ delivery to just a few trials per day. Even with fewer trials, ADO shows superior reliability and precision in behavioral parameter estimation than non-ADO methods [29].

We focused on two main objectives: first, we aimed to identify computational and psychological markers predictive of short-term (day-to-day smoking) and long-term (post-treatment abstinence) outcomes. Second, in more exploratory analyses, we aimed to test the efficiency of ADO-powered computational markers for the latter by determining the minimum amount of data required for reliable prediction. For this purpose, we varied the informational window for prediction (i.e., by binning data collection weeks) to determine if a more limited data collection regimen could be used in future studies to infer end-of-treatment smoking cessation status 5-6 weeks later. These goals are particularly beneficial to achieve because identifying daily predictors of smoking behavior highlights the potential for adjusting treatment plans dynamically to improve outcomes, while predicting longer-term abstinence could support improved treatment selection and participant stratification, together offering valuable insights for individualized prognosis. Further, determining whether longer-term outcomes can be reliably predicted from early or reduced assessment windows has important practical implications for developing lower-burden monitoring approaches.

## Methods

### Participants

We recruited daily cigarette smokers (*N* = 143) enrolled in a 5-6 week medication-assisted smoking cessation program that included weekly counseling and prescription of varenicline, bupropion, or nicotine replacement therapy (NRT; gum, candy, or patch), as needed. All participants provided written informed consent prior to enrollment, including consent to screening session and study participation. Participants underwent a screening session prior to initiating treatment to determine if they met the following criteria: (1) smoked >5 cigarettes/day in the past year; (2) had no current psychiatric disorder diagnoses other than tobacco use disorder, or used psychiatric medications within the past year, as assessed by the Structured Clinical Interview for DSM-5 Disorders: Clinical version (SCID-5) [30]; (3) had a negative urine drug screen (for stimulants, opioids, and cannabis) and breath alcohol test; and (4) abstained from smoking cigarettes for ≥8 h prior to study visits, verified via exhaled CO levels <12 ppm [31]. All procedures were approved by the Institutional Review Board of Seoul National University. Data were collected from March 2019 to February 2023, with a temporary suspension in 2020 due to the COVID pandemic. Data collection resumed in January 2021.

### Psychological state surveys and self-reported smoking

Following screening (pre-clinic visit), participants installed a study app on their smartphones. Throughout the treatment period, the app notified participants three times daily (9 am, 2 pm, 7 pm) to rate their current psychological state using a 0-100 scale. Participants were instructed to report their current mood, stress, depression, anxiety level, and craving since the last prompt. They were instructed to complete the surveys within 3 h of notification. In addition, participants reported their cigarette consumption and whether they had taken their prescribed smoking cessation medication since the last prompt.

In addition, during pre- and post-clinic visits, participants reported their average daily cigarette use over the past week in four categories (0, ≤5, >5 but <10, and ≥10 cigarettes smoked). They also completed the Fagerström test for nicotine dependence (FTND) to assess smoking severity.

### Decision-making tasks

Throughout the treatment period, the study app also notified participants once daily to complete two decision-making tasks. For both the delay discounting and CRA tasks, participants were instructed to choose their preferred option between two choices, with the expectation of a hypothetical monetary reward. See Supplement for additional details. We used ADO, which is a machine-learning method originating from active learning [32], to optimize experimental variables in real-time, every time participants completed the tasks. We used a Python library called ADOpy [33] to incorporate ADO into the task generated with a Python library (PsychoPy).

*Computational model of the Choice under risk and ambiguity (CRA) task* [27]

**Fig. 2:**
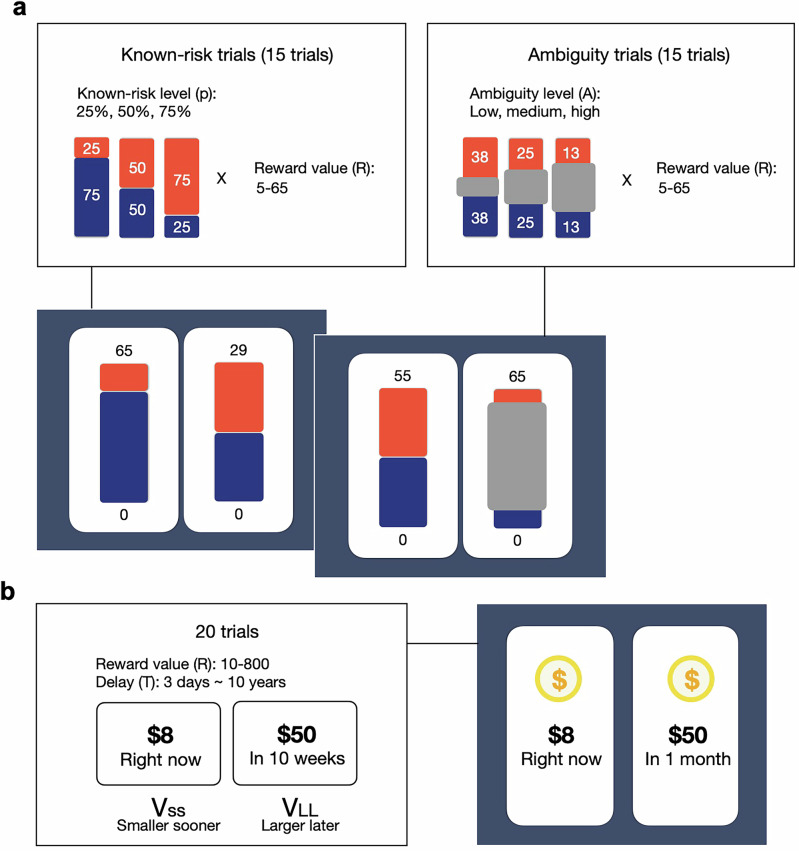
ADO-powered decision-making tasks are delivered daily via a smartphone app. **a** Choice under Risk and Ambiguity (CRA) task. The task consisted of two types of trials: known-risk and ambiguity. In each trial, participants choose between two options (one with a fixed 50% probability of winning a non-zero reward amount vs. one varying in probability of reward). Choices were based on the amount of hypothetical reward on offer (displayed on each trial on the top and the bottom of each colored box) and its probability (indicated by the size of each colored box). During ambiguous trials, a grey box hid the exact probability of getting a reward in one of the two options. Choice behavior on the CRA task was used to estimate a participant’s day-level risk (α) and ambiguity tolerance (-β) parameters. **b** Delay discounting (DD) task. Participants chose between two options, a smaller, sooner reward and a larger, delayed reward; these choices were used to estimate their day-level delay discounting rate (k). Note that dollar signs were used here to aid understanding, but participants were presented with Korean won on a similar scale.

We used an ADO-powered CRA task to evaluate responses to risk and ambiguity, with known and unknown (ambiguous) reward probabilities (Fig. 2a). The CRA task consisted of known-risk trials and ambiguous trials. In known-risk trials, participants marked their preference between two options with the explicit probability (*p*) of reward (*R*) and the amount of reward (range 5-65). Whereas, in ambiguous trials, the probability of reward was partially hidden and therefore rendered ambiguous, using three levels of occlusion, i.e., ambiguity (*A*): 24%, 50%, or 74%. No outcomes (feedback) were shown during the task.

We modeled behavior using a modified utility model, as in prior studies [27], where the utility (*U*) of each option was based on reward probability (p), reward amount (*R*), ambiguity (*A*), and individual subject- (and day-) specific risk tolerance (α) and ambiguity sensitivity (β), as follows:

1 $${U}_{{option}}=[p-\beta (\frac{A}{2})]* {R}^{\alpha }$$

In each trial, two types of options were presented: one option with fixed reward probability of 0.5 and the other option with varying reward probability (either 0.25, 0.5, or 0.75). Therefore, the utility of each option can be rewritten as follows, where ${U}_{{Fix}}$ and ${U}_{{Var}}$ denote the utility of fixed option and variable option.

2 $$\begin{array}{c}{U}_{{Fix}}=0.5* {R}^{\alpha }\\ {U}_{{Var}}=[p-\beta (\frac{A}{2})]* {R}^{\alpha }\end{array}$$

The probability of choosing the variable, i.e., the risky or ambiguous option (${\Pr }_{{Var}}$), was computed as the difference in the utilities of the fixed-probability (${U}_{{Fix}}\left)\right.$ and variable (${U}_{{Var}}\left)\right.$ options. The final choice probability was computed with a softmax function of utility value difference with the inverse temperature γ, which captured choice stochasticity (Eq. 3).

3 $${Pr }_{{Var}}=\frac{1}{1+\exp [-\gamma \left({U}_{{Var}}-{U}_{{Fix}}\right)]}$$

In this model, a risk tolerance parameter (α)<1 indicates risk aversion, α > 1 indicates risk seeking, and α = 1 indicates risk neutrality. Similarly, an ambiguity sensitivity parameter *β* > 0 indicates ambiguity aversion, *β* < 0 indicates ambiguity tolerance (bearing with the unknown probability), and *β* = 0 ambiguity neutrality. For ease of interpretation, we report -*β*, with higher -*β* parameter values indicating greater ambiguity tolerance.

*Computational model of the Delay discounting task* [24]

The delay discounting task required participants to choose between a larger-later (LL) and smaller-sooner (SS) monetary reward, with random positioning on the screen (Fig. 1C). We used the 20-trial delay discounting task powered by ADO, shown to robustly estimate discount rates in our prior work [29]. No outcomes (feedback) were shown during the task.

Using the hyperbolic model (Eq. 4) [34], we estimated the delay discounting rate (k), log-transformed to log(k) to address skewness in the distribution of k values. Larger log(k) values indicate greater temporal discounting rate, or choice impulsivity. The subjective value (V) of each option was calculated based on the reward (R) and delay in time (T) to reward on offer (Eq. 3).

4 $$V=R* \frac{1}{1+{kT}}$$

Value of LL and SS option could be re-written as Eq. 5, where the reward is multiplied by a discounting factor and which decreases as a function of delay (T) and the discounting rate (*k*).

5 $$\begin{array}{c}{V}_{{LL}}={R}_{{LL}}* \frac{1}{1+{kT}}\\ {V}_{{SS}}={R}_{{SS}}* \frac{1}{1+{kT}}\end{array}$$

Same as the model for the CRA task, the final choice probability was computed with a softmax function of value difference with the inverse temperature τ, reflecting choice sensitivity. Specifically, the probability of choosing the LL option over the SS option is denoted as Pr (LL) in Eq. 6.

6 $${\Pr }_{{LL}}=\frac{1}{1+\exp [-\tau \left({V}_{{LL}}-{V}_{{SS}}\right)]}$$

### Model fitting

We estimated the model parameters using Rstan with the Markov chain Monte Carlo (MCMC) sampler (i.e., Hamiltonian Monte Carlo algorithm). For the parameter estimation, 4 chains, 4000 iterations with 2000 warmups were used. To ensure reliable and rapid parameter estimation, we evaluated convergence diagnostics with R-hat values below 1.01, with most model fits converging within 1-2 attempts (Table S6), indicating stable and well-mixed MCMC chains (Fig. S5). To assess parameter identifiability, we conducted parameter recovery analyses under the same task structure as the empirical study, and we found a reliable recovery rate (*r* = 0.71–0.96, Fig. S6). In addition, parameters with estimated z-scores exceeding ±1.96, based on the 95% Highest Density Interval (HDI), were considered grounds for exclusion. However, no participants met this criterion, and therefore, none were excluded from further analysis.

### Short-term (day-to-day) prediction of smoking behavior

**Fig. 3:**
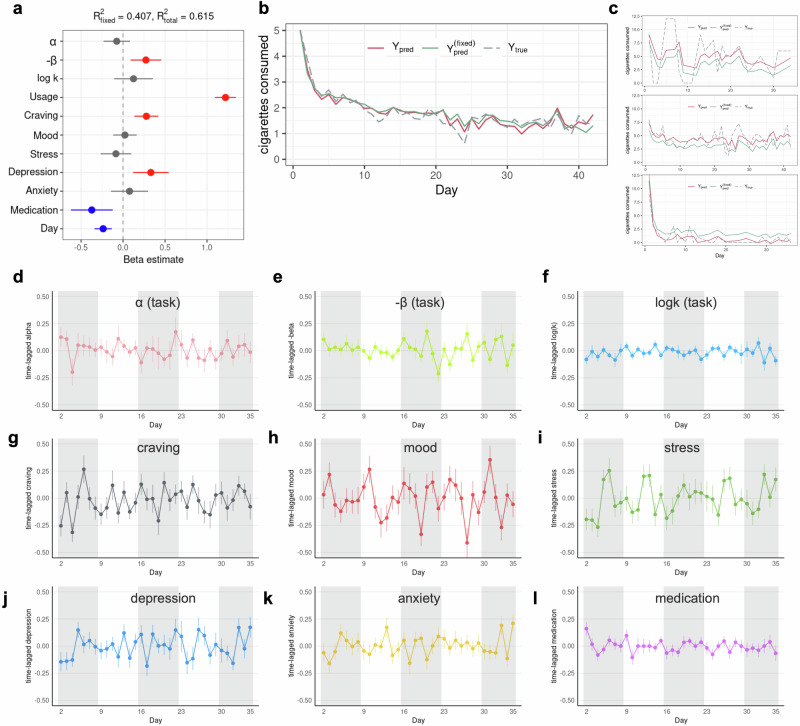
Daily predictors of smoking behavior. **a** Coefficient plot of the winning time-lagged prediction model predicting next-day number of cigarettes smoked from current day self-report and task-related variables (the last model [#29] in Table S1). Colored dots indicate variables with statistically significant coefficients. Error bars indicate 95% confidence interval. **b** Model predictions and actual cigarette consumption over time. The plot compares the observed cigarette consumption (${Y}_{{true}}$) with the predicted values from a mixed-effects model (${Y}_{{pred}}$) and its fixed-effects component (${Y}_{{pred}}^{{fixed}}$). It suggests that the model is not only capable of accounting for day-to-day variations in smoking but also follows broader patterns of reduction in cigarette usage across the treatment days. This similarity underscores the model’s utility for understanding smoking behavior dynamics, particularly for predicting future consumption based on prior variables. **c** Predicted and true cigarette consumption for a single example individual with high variability in cigarette consumption (top plot), a single example individual with low variability in cigarette consumption (bottom plot), and average of a random subset of 10 individuals (middle plot). **d**–**l** Time-lagged average z-scored values for each predictor (alpha, -beta, log(k), craving, mood, stress, depression, anxiety; **d–k**), and time-lagged average values for medication intake (**l**). Scores for each variable are computed as the discrepancy between Day_t+1_ and Day_t_, and the error bars indicate standard error.

To identify factors that can predict proximal (short-term) smoking behavior, we conducted a time-lagged analysis using a linear mixed-effect model (*lme* package in R). Independent variables included task parameters (log(k), α, and -β) and the self-reported psychological states (anxiety, craving, depression, stress, and mood) from the current day (t) to predict cigarettes smoked on the next day (t + 1)(Eq. 7). *Day* (i.e., elapsed days at the clinic) was added as a fixed effect to account for the non-linear decline in cigarette use over time (Fig. 3B). Between-subject variability was modeled as a random effect. In addition, all predictors were z-scored within individuals to ensure person-centered estimation, allowing us to investigate whether within-person day-to-day changes in psychological or behavioral variables predicted smoking behavior the following day.

7 $${y}_{t+1}={x}_{t}^{1}+{x}_{t}^{2}+...\,{x}_{t}^{n}+\left(1|{subject}\right)$$

To assess model performance, we used a 3-fold cross-validation at the participant level. In each repetition, the dataset was randomly divided into three subsets (folds). Specifically, the model was first trained on two folds and tested on the remaining fold, and this was repeated such that each fold served once as the test set. Importantly, while the model preserved temporal directionality, such that predictors measured at day *t* were used to predict smoking behavior at day *t* + *1*, the primary cross-validation procedure did not explicitly enforce strict temporal partitioning within individuals. As a complementary analysis, we conducted time-aware (forward-chaining) cross-validation, training on earlier sessions and testing on later ones; results are reported in Table S2. Model performance was evaluated using R^2^ (explained variance) and mean squared error (MSE), averaged across the three test folds to obtain a single cross-validated estimate per repetition. To reduce dependence on a specific data partition, the entire cross-validation process was repeated 100 times with different random fold assignments. Final performance metrics were computed by averaging R^2^ and MSE across iterations. The best-performing model was selected as the model yielding the highest mean R^2^ and the lowest mean MSE across repetitions on the test set. To further investigate day-to-day variability in the predictive variables, we quantified intraclass correlation (ICC) and root mean square of successive differences (RMSSD) using *the irr* package and the *EMAtools* package in R.

### Long-term (post-treatment) prediction of smoking cessation

To predict long-term smoking cessation after treatment, we ran penalized logistic regression with 10-fold cross-validation (*easyml* package in R). In order to include multiple correlated features into the prediction model, we implemented an Elastic Net model. The model includes two hyperparameters: the mixing parameter (alpha), which determines the relative contribution of Lasso and Ridge regularization, and the strength parameter (lambda), which controls the overall degree of coefficient shrinkage. We evaluated 10 candidate mixing parameters (alpha) values ranging from 0 (pure Ridge) to 1 (pure Lasso) to mix the two regularization methods. For each alpha and for each fold of the 10-fold cross-validation, lambda was selected by internal cross-validation performed only within the training set. Predictive performance was quantified using the area under the curve (AUC). The 10-fold cross-validation procedure was repeated 100 times, yielding 1000 total train-test evaluations, and AUC values were averaged across the 1000 iterations. This was to ensure a robust estimation of the AUC values for different train-test splits under a limited number of samples [35]. The alpha value yielding the highest mean AUC (alpha = 0.6) is reported in the main text.

In all the models, the dependent variable was defined as a binary outcome, indicating successful maintenance of smoking cessation after the clinic. Success was defined as maintaining cessation for one month, verified by an on-site exhaled CO test result below 5 ppm. Main predictors included EMA survey responses and decision-making model parameters (log(k), α, and -β). To control for potential confounding effects, we included years of smoking history and weekly clinic participation rate, as well as age and sex, as covariates in the analysis reported predicting smoking cessation status.

To examine the minimum data required to predict smoking cessation status, we tested 14 candidate models (Models A-N), representing different combinations of task parameters and survey data and data aggregation windows (e.g., first week vs. all available weeks; Tables S4–5). We employed four distinct data preprocessing methods for each modality (task and survey). In the first method, a single datapoint from the first week was randomly selected to represent that week. In the second method, the mean of all available data points from the first week was used. The third method involved calculating the mean of all data points collected throughout the entire data collection period (5–6 weeks) and using this value. The final method involved randomly selecting one datapoint from each week across the entire data collection period, averaging these values over 100 iterations, and using the resulting average for the prediction.

## Results

### Participant characteristics

A total of 79 participants were included in the final analysis (Fig. S1). Participants provided an average of 23.3 daily observations across the study period, indicating relatively dense longitudinal sampling. Data availability across participants and days is visualized in Fig. S4. For details of the screening criteria, refer to the Methods section. Participants were ad-hoc grouped based on their end-of-treatment smoking status: the “quit group” (4 weeks of self-reported abstinence and post-clinic exhaled Carbon Monoxide (CO) ≤ 5ppm) and the “failed group”. The quit and the failed groups did not significantly differ in their demographic and baseline (pre-clinic) smoking-related characteristics, except for a longer smoking duration in the quit group (${{Mean}}_{{quit}}$ = 4.4 years, ${{SD}}_{{quit}}$ = 3.82, ${{Mean}}_{{fail}}$ = 7.7 years, ${{SD}}_{{fail}}$ = 4.25, *p* = 0.001). In addition, the two groups showed a marginal difference in clinic participation, which was computed as a ratio of the number of available clinic visits during the treatment program and the individual’s total number of actual visits (${{Mean}}_{{quit}}$ = 71.15%, ${{SD}}_{{quit}}$ = 25.63, ${{Mean}}_{{fail}}$ = 58.15%, ${{SD}}_{{fail}}$ = 28.61, *p* = 0.061) (Table 1). To control for potential confounding effects, we included years of smoking history and clinic participation rate, as well as age and sex, as covariates in the analysis reported predicting smoking cessation status.

**Table 1:** Demographic information of the sample, split into the failed vs. quit (successful) smoking cessation groups. CO=Exhaled breath Carbon Monoxide level.

Characteristic	Failed to Quit (*N* = 60)		Successfully Quit (*N* = 19)		t-value	*p*-value
	Mean (N)	SD (%)	Mean (N)	SD (%)		
Age	26	3.84	26.3	3.68	-0.29	0.777
Sex: Male	49	(82%)	17	(89%)		0.7
Smoking duration (years)	4.4	3.82	7.77	4.25	–4.57	0.001
CO: screening (ppm)	4.12	3.29	1.68	0.82	3.47	0.001
CO: pre-clinic (ppm)	4.12	3.31	2.63	2.06	2.33	0.024
CO: post-clinic (ppm)	4.28	3.74	2.37	1.16	5.23	<0.001
Clinic participation (%)	58.15	28.61	71.15	25.63	–1.53	0.061
App participation (%)	71.6	23.2	68	20.5	0.52	0.523
FTND	2.53	2.04	2.05	1.51	1.51	0.275

### Short-term (day-to-day) prediction of smoking behavior

We evaluated 29 candidate models to identify predictors of next-day cigarette consumption using time-lagged mixed-effects regression (Table S1). Under standard cross-validation, the most comprehensive model incorporating both task-derived computational parameter estimates and EMA variables achieved the best predictive accuracy compared to other models (Model 29: R²_test_ = 0.552, MSE = 4.845). This model revealed significant contributions from the number of cigarettes smoked, cigarette craving, and depression level on the current day, which all positively predicted the number of cigarettes smoked on the next day (Fig. 3a). Medication intake, by contrast, was associated with decreased smoking amount on the next day. Note that, as all participants were enrolled in the smoking cessation clinic, which indirectly reflects their motivation to quit smoking, the mean amount of smoking decreased significantly over the course of the study (Fig. 3b). To assess robustness under a more stringent prospective prediction framework, we conducted time-aware (forward-chaining) cross-validation (Table S2). Under this scheme, usage and craving emerged as the dominant proximal predictors (Model 18, R²test = 0.483), with additional EMA variables such as mood, stress, and anxiety contributing little further improvement (Model 23, R²test = 0.466). The addition of computational task parameters to the full EMA model recovered some of this loss (Model 26, R²test = 0.479), representing a small but non-negligible improvement over EMA measures alone. Overall, the contribution of task parameters retained modest incremental predictive value beyond self-report measures.

Beyond self-report predictors, we also found a significant contribution of the ambiguity tolerance parameter (-β) in predicting increased smoking amount on the next day. In contrast, the other model parameters, risk tolerance (α) and discounting rates (log(k)), did not contribute significantly (Table S3). A higher ambiguity tolerance (-β) parameter suggests individuals more comfortable with uncertainty may be more willing to smoke despite the unknown risk for relapse and treatment failure, consistent with previous findings in other substance use disorders [14]. This effect remained even after controlling for medication use (Table S1). Further, even after controlling for the primary variance-driving factor of prior-day cigarette usage, both the ambiguity tolerance parameter and craving remained significant positive predictors in the model (Fig. S3). This outcome suggests that these factors contribute uniquely to cigarette consumption risk, independent of prior-day usage effects, which underscores the robustness of the result. Specifically, the full model, which included both task and survey variables, predicted smoking patterns well in individuals with both high and low variability in their cigarette consumption (Fig. 3b, c), demonstrating its potential to capture daily fluctuations in individuals with a range of smoking behaviors.

The task parameters had higher ICC values than survey responses (Table S3), indicating a better relative consistency. In addition, there was a moderate level of within-person variability captured by RMSSD scores in the task parameters, potentially reflecting changes tied to their clinical condition (i.e., smoking).

### Long-term (post-treatment) prediction of smoking cessation

For the long-term (post-treatment) prediction of smoking cessation, we aimed to identify and evaluate the efficiency of ADO-powered computational markers based on predictive accuracy. We took the mean of 1000 iterations of the area under the curve (AUC) value as the measure of model accuracy (for more details, see “Methods”).

Models incorporating data from the full study period achieved the highest predictive accuracy (Model K, mean AUC = 0.83, Tables S3-4**;** Model N, mean AUC = 0.82). However, these should be interpreted with caution as upper-bound estimates of predictive performance, as these models include information temporally proximal to the outcome.

We tested whether we could achieve comparable predictive accuracy using a shorter follow-up window. In particular, we tested whether data from just the first one or two weeks of data collection could be sufficient to flag at-risk/resilient individuals, thus allowing for early, more targeted interventions aimed at obtaining a higher probability of treatment success. A model using only the first week of task data (Model B, mean AUC = 0.74) performed similarly to models based on survey data (Model F, mean AUC = 0.73) or both task and survey data, which demonstrated the highest accuracy at this timeframe (Model J, mean AUC = 0.76). Extending data collection beyond the first week (Model C, mean AUC = 0.74) or adding more data within a shorter timeframe (Model D, mean AUC = 0.73) did not improve prediction further. These results suggest that task-based computational markers from the first week of treatment can effectively predict cessation outcomes at the end of treatment, 4–5 weeks later.

**Fig. 4:**
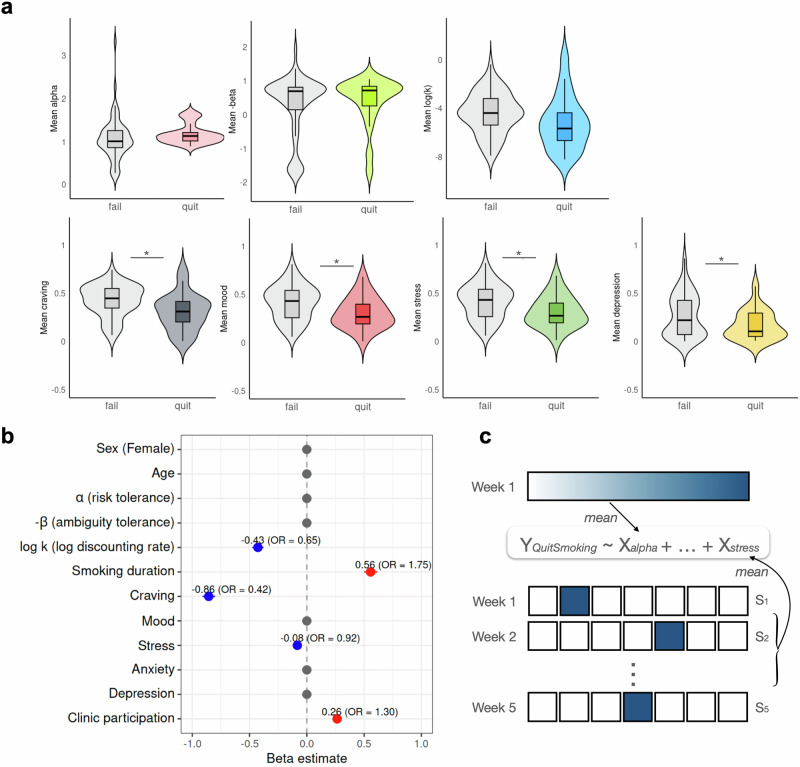
Computational task-based and psychological markers of successful smoking cessation post-treatment. **a** Group comparison of the computational task-based (top row) and clinical predictors (bottom row) across the fail (relapse) and quit groups. **b** Results of linear logistic regression predicting the likelihood of successful smoking cessation following 5-6 weeks of treatment. The labels on the left represent the input variables, for which the mean values collected during the first week of follow-up were included as predictors in the model (Model J, Table S5). A lower discounting rate (k), lower craving and stress levels, along with a longer history of smoking and active participation in the smoking cessation program, were predictive of successful treatment outcomes. OR=Odds Ratio. **c** Illustration of preprocessing input variables for the winning model (Model J). For computational markers, mean values of the first week’s data were taken and for subjective responses, mean values of the randomly selected days from every week’s data were taken as predictors of the linear regression model.

Key predictors of cessation success across these predictive models included lower discounting rates (log(k)), lower craving and stress, a longer smoking history, and greater treatment engagement (Fig. 4). The association between lower delay discounting and successful cessation aligns with prior research on impulsivity as a strong relapse factor, as higher delay discounting rates are often associated with impulsivity and difficulty with delaying gratification [36, 37]. Additionally, participants with a longer smoking history and greater engagement with treatment sessions were more likely to succeed in quitting smoking, potentially reflecting increased motivation to quit among long-term smokers. A marginal correlation was also observed between smoking history and daily compliance with the study protocol (*r* = 0.2, *p* = 0.08).

While a longer follow-up window improves prediction as a rule of thumb, we show that computational markers derived from a more limited data collection period (e.g., the first week) still provide practical insights that can be leveraged in treatment programs, especially when extensive data collection is not feasible.

## Discussion

Nicotine addiction is a chronic, relapsing condition, highlighting the need to identify dynamic predictors of smoking behavior across both short and long timescales. Although theory and initial empirical work in other addictions suggest that frequent sampling is needed to capture fluctuations in latent neurocognitive processes, such approaches have rarely been applied to smoking, particularly at clinically relevant, daily resolution. Traditional methods, such as EMA—based solely on survey data—or computational modeling requiring lengthy tasks also face significant feasibility and precision challenges in clinical settings.

To overcome these challenges, we combined ADO-powered decision-making tasks with EMA to efficiently collect computational and psychological markers on a daily basis over 5–6 weeks of a smoking cessation program, enabling both within-person prediction of daily smoking behavior and prospective classification of post-treatment cessation status for held-out participants using as little as one week of data. This innovative approach provided an efficient, low-burden framework for capturing the cognitive factors influencing smoking cessation outcomes.

At the day-to-day level, ambiguity tolerance emerged as a significant predictor alongside craving and stress of next-day cigarette consumption, even after controlling for current-day smoking and medication use. Elevated ambiguity tolerance was associated with increased risk of returning to smoking, consistent with prior research in other substances [14]. Although rarely studied in treatment outcomes research, taken together with prior work, ambiguity tolerance may represent a clinically actionable marker for identifying (and potentially mitigating) periods of heightened substance use vulnerability. Interestingly, the best model predicted both day-to-day smoking and overall trends in cigarette intake during treatment, indicating potential utility for monitoring ongoing risk states and informing just-in-time interventions. Although prior-day smoking behavior and craving accounted for much of the predictive variance, the addition of ADO-derived computational parameters provided (albeit modest) predictive value beyond EMA self-report measures alone. Crucially, these parameters capture latent decision-making processes that EMA surveys do not directly index, offering a complementary and mechanistically interpretable window into the drivers of smoking behavior. We note that the day-to-day associations in the current study warrant interpretation as reflecting within-person fluctuations relative to each individual’s characteristic (baseline) state across the treatment period. Future work employing a longer or denser follow-up period may consider alternative centering approaches, such as training-set-only centering, to more cleanly assess predictive relationships in day-to-day analyses and further strengthen the strictly prospective interpretation of these findings.

In contrast, a distinct decision-making process—delay discounting—predicted longer-term outcomes. Specifically, models incorporating average levels of task-based and survey measures across the study period best predicted post-treatment cessation success: individuals with lower overall discounting rates, lower craving and stress, higher treatment adherence, and longer smoking histories were more likely to maintain abstinence. Notably, delay discounting estimates obtained only during the first week of treatment predicted later cessation success, with accuracy comparable to models based on more extensive data or survey responses. Our findings replicate prior findings linking delay discounting to smoking cessation outcomes [26, 38] while extending these to the specific context of ADO-powered designs and tasks administered in the natural environment to anticipate overall consumption trends from brief assessments in the early treatment period, which could prove useful for early identification of relapse risk and guiding adjustments in treatment intensity to improve long-term cessation outcomes.

An open empirical question is why delay discounting predicted longer-term cessation outcomes, whereas ambiguity tolerance predicted short-term, daily smoking. This dissociation is unlikely to reflect differences in temporal variability, given similar RMSSD and ICC metrics. Instead, these parameters likely capture distinct aspects of addiction-relevant decision-making: delay discounting indexes the degree to which individuals are future-oriented, while ambiguity tolerance captures an optimistic or permissive response to uncertainty. Accordingly, higher discounting rates could indicate a tendency to devalue the delayed health benefits of quitting smoking relative to the immediate reinforcing effects of nicotine [39], shaping the ability to maintain abstinence over time. Higher ambiguity tolerance could instead reflect increased willingness to accept the uncertain health risks of smoking or the possibility of treatment failure, thereby influencing cigarette consumption, particularly under fluctuating internal states and contexts. Because ambiguity tolerance reflects sensitivity to uncertainty distinct from known risk [40, 41], it may be more relevant for short-term behavioral regulation rather than long-term cessation outcomes.

Although the mechanisms underlying this dissociation remain unclear, the results underscore the importance of intra- and inter-individual differences in decision-making across stages of smoking cessation and the utility of computational approaches for parsing this heterogeneity [42]. By capturing dynamic, value-based decision processes in real time, this framework holds promise for advancing ecological and predictive understanding beyond nicotine addiction, extending to other psychiatric conditions characterized by symptom fluctuation and uncertainty in prognosis.

### Limitations

Our sample mostly comprised young male smokers screened for other substance use and psychiatric disorders, enhancing experimental control but potentially limiting generalizability. Nevertheless, this population remains critical to study, as over half of adult smokers begin before the age of 18, and early smoking onset substantially increases lifelong addiction risk [43]. Future studies that broaden sampling could enhance the applicability of findings. In addition, some potentially relevant covariates (e.g., household smoking exposure, prior quit attempts) were not available in the current dataset and could be incorporated in future studies to further broaden understanding.

## Conclusion

To our knowledge, this is the first study to combine self-report and task-based data to predict smoking behavior at this fine-grained (daily) level during treatment. We found that just one week’s worth of data was sufficient to achieve high predictive power for anticipating post-treatment smoking cessation outcomes. This has important implications not only for the potential to reduce participant burden but also for enhancing the feasibility of integrating such assessments into real-world clinical settings, where there are practical constraints with participant engagement and retention in studies involving participants with psychiatric disorders.

Together, our results highlight the promise of integrating real-time responses with ADO-powered cognitive tasks to enhance the precision of behavioral health predictions. This approach is both reliable and efficient in capturing individual differences while minimizing participants’ time and cognitive burden and yielding rich, actionable clinical insights. We hope these results offer novel predictive insights and open possibilities for more effective and precise treatments for nicotine addiction and related psychiatric conditions, fostering the development of individually-tailored interventions in clinical practice.

## Supplementary information

Supplementary Materials

## Data Availability

All the codes for the analysis and data can be found online at https://github.com/CCS-Lab/SmokingPred_ADO.

## Code availability

All the codes for the analysis and data can be found online at https://github.com/CCS-Lab/SmokingPred_ADO.
