# Supplementary material for: Rapid and reliable computational markers of decision-making for predicting daily smoking behavior and smoking cessation treatment outcomes
Source: Neuropsychopharmacology. 2026 May 29;51(10):1881–90. doi: 10.1038/s41386-026-02448-5 (PMC13487167; doi:10.1038/s41386-026-02448-5)
Supplement: Supplementary file 1 [file 41386_2026_2448_MOESM1_ESM.docx]

**Supplementary information**

**Participation bonus criteria**

Participants were compensated with a financial bonus in accordance with their app participation, where they were incentivized for consistent participation in the experiment. “Consistent participation” was defined as completing at least 2 out of the 3 daily assigned surveys and both tasks on a given day. During the 5-6 weeks of the experiment, participants who met the criteria for consistent participation on at least 5 days in a given week received $15 (KRW 20,000) for that week, while those who met the criteria on 3 to 5 days in a given week, received $7.50 (KRW 10,000). Additionally, participants who consistently participated for at least 5 days a week across the full 5-6-week study period were given an extra $15 (KRW 20,000). The total maximum monetary compensation for participation in the app-based tasks (assuming 5 weeks) was $90 (KRW 120,000).

**Computational modeling of decision-making tasks**

We used uniform prior distributions and grid-based parameter estimation to update prior distributions of model parameters (from standard cognitive models of these tasks; see below) into posterior distributions, based on Bayes’ rule.

Specifically, for the choice under risk and ambiguity task, it initially consisted of 60 trials (N=22, in 2019). However, analysis showed 30 trials were sufficient to estimate model parameters (**Fig. S2**), so the number was reduced for subsequent data collection. The original parameter boundary settings for the ADO-based CRA task collected in 2019 (N=22) differed from those of other datasets. During the initial data collection, the permissible parameter range for ⍺ (risk tolerance) was [0, 1] and for -β (ambiguity tolerance) it was [-1, 1]. Because the parameters were often hitting the ceiling or floor of these ranges, we expanded the grid space of parameters to [0, 5] for ⍺ and [-3, 3] for -β.

For the delay discounting task, The delay in time (T) varied from days to years (3 days, 5 days, 1 week, 2 weeks, 3 weeks, 4 weeks, 6 weeks, 8 weeks, 10 weeks, 12 weeks, …, 5 years, and 10 years) and the amount of reward (R) was fixed to $800 for the larger and later option, while the grid space was defined within a range of (10, 800).

All the parameters were estimated by fitting individual Bayesian models using Rstan 2.26.3 in R 4.2.1. This was to avoid further risk of skewed estimation or limited interpretative power of the model. We estimated the model parameters using Rstan with the Markov chain Monte Carlo (MCMC) sampler (i.e., Hamiltonian Monte Carlo algorithm) to mitigate the risk of inconsistency in parameter estimation. Parameters estimated with ADO and MCMC were highly correlated with each other (e.g., for log(k), correlation coefficient between MCMC and ADO estimates was 0.989).

**Supplementary Figures & Tables**

**Fig. S1.** **CONSORT diagram for participants inclusion.** Flow diagram showing the number of participants included/excluded at each recruitment and analysis step.

**Fig. S2.** **(A)** Test-retest reliability for alpha and beta parameters in the CRA task, using 30 and 60 trials. It was computed by the Pearson correlation between values from two consecutive days. The x-axis labels represent the two days of which the correlation was computed. **(B)** The distribution of parameter values for both 30 and 60 trials is shown.

**Fig. S3.** Mixed-effects model predicting the residual variance in cigarette usage after controlling for the primary usage variable. The model was run on residual cigarette usage (usage on day *t+1* after regressing out usage on day *t*) and includes all other predictors of interest, excluding the primary usage variable that accounted for a large portion of the variance in the original model. The beta estimates (fixed effects) for each predictor are displayed with 95% confidence intervals. Significant predictors (p<0.05) are shown in red.

**Fig. S4. Availability of daily observations across participants and study days.** Participants provided an average of 23.3 daily observations, with variability reflecting typical fluctuations in compliance rather than systematic dropout.

**Fig. S5.** **Distribution of R-hat values across parameters.** Distributions of the R-hat values are shown for each model parameter across subjects. Separate panels correspond to parameters from the CRA (α, β, log γ) and DDT (log k, log τ) tasks. The dashed vertical line indicates the convergence threshold (R-hat = 1.01). Across all parameters, most R-hat values were tightly concentrated near 1.00 and remained below the threshold, indicating good convergence of the MCMC chains and reliable parameter estimation.

**Fig. S6.** Parameter recovery analysis. Parameter recovery was assessed using simulated datasets (N=100 subjects). (a) Parameters estimated from the Delay-Discounting task. (b) Parameters estimated from the Choice Under Risk and Ambiguity task.

|  |  | Train | | | Test | | |
| --- | --- | --- | --- | --- | --- | --- | --- |
| # | ${IV}_{fixed}$ | $R^{2}$ | MSE | $\Delta MSE$ | $R^{2}$ | MSE | $\Delta MSE$ |
| 1 |  | 0.526 | 5.134 | 0.967 | 0.461 | 5.832 | 0.987 |
| 2 | 𝛼 | 0.528 | 5.120 | 0.953 | 0.460 | 5.847 | 1.002 |
| 3 | 𝛼, Day | 0.549 | 4.886 | 0.718 | 0.484 | 5.583 | 0.738 |
| 4 | −𝛽 | 0.527 | 5.129 | 0.961 | 0.461 | 5.839 | 0.994 |
| 5 | −𝛽, Day | 0.549 | 4.887 | 0.719 | 0.486 | 5.562 | 0.717 |
| 6 | log k | 0.527 | 5.122 | 0.954 | 0.464 | 5.806 | 0.961 |
| 7 | log k, Day | 0.548 | 4.899 | 0.731 | 0.487 | 5.561 | 0.716 |
| 8 | 𝛼, −𝛽, log k | 0.529 | 5.104 | 0.936 | 0.462 | 5.830 | 0.985 |
| 9 | 𝛼, −𝛽, log k, Day | 0.550 | 4.880 | 0.712 | 0.485 | 5.578 | 0.733 |
| 10 | Usage | 0.599 | 4.347 | 0.180 | 0.540 | 4.975 | 0.130 |
| 11 | Usage, Day | 0.605 | 4.277 | 0.110 | 0.545 | 4.917 | 0.072 |
| 12 | Usage, 𝛼 | 0.599 | 4.342 | 0.175 | 0.539 | 4.983 | 0.138 |
| 13 | Usage, 𝛼, Day | 0.605 | 4.278 | 0.110 | 0.546 | 4.908 | 0.063 |
| 14 | Usage, −𝛽 | 0.600 | 4.336 | 0.168 | 0.539 | 4.993 | 0.148 |
| 15 | Usage, −𝛽, Day | 0.606 | 4.264 | 0.096 | 0.545 | 4.918 | 0.073 |
| 16 | Usage, log k | 0.600 | 4.336 | 0.169 | 0.536 | 5.014 | 0.169 |
| 17 | Usage, log k, Day | 0.604 | 4.284 | 0.116 | 0.546 | 4.905 | 0.059 |
| 18 | Usage, Craving | 0.603 | 4.299 | 0.132 | 0.544 | 4.926 | 0.081 |
| 19 | Usage, Craving, Day | 0.608 | 4.246 | 0.078 | 0.549 | 4.884 | 0.038 |
| 20 | Usage, Craving, 𝛼, −𝛽, log k | 0.605 | 4.280 | 0.113 | 0.544 | 4.935 | 0.090 |
| 21 | Usage, Craving, 𝛼, −𝛽, log k, Day | 0.609 | 4.237 | 0.069 | 0.550 | 4.867 | 0.022 |
| 22 | Usage, Craving, Mood, Stress, Depression, Anxiety, Medication | 0.608 | 4.243 | 0.075 | 0.546 | 4.903 | 0.058 |
| 23 | Usage, Craving, Mood, Stress, Depression, Anxiety | 0.607 | 4.253 | 0.086 | 0.543 | 4.932 | 0.087 |
| 24 | Usage, Craving, Mood, Stress, Depression, Anxiety, Day | 0.612 | 4.204 | 0.036 | 0.551 | 4.858 | 0.013 |
| 25 | Usage, Craving, Mood, Stress, Depression, Anxiety, Medication, Day | 0.614 | 4.177 | 0.009 | 0.550 | 4.864 | 0.019 |
| 26 | Usage, Craving, Mood, Stress, Depression, Anxiety, 𝛼, −𝛽, log k | 0.608 | 4.240 | 0.073 | 0.545 | 4.919 | 0.074 |
| 27 | Usage, Craving, Mood, Stress, Depression, Anxiety, Medication, 𝛼, −𝛽, log k | 0.610 | 4.225 | 0.057 | 0.545 | 4.918 | 0.073 |
| 28 | Usage, Craving, Mood, Stress, Depression, Anxiety, 𝛼, −𝛽, log k, Day | 0.613 | 4.191 | 0.024 | 0.552 | 4.848 | 0.002 |
| 29 | Usage, Craving, Mood, Stress, Depression, Anxiety, Medication, 𝛼, −𝛽, log k, Day | 0.615 | 4.168 | 0.000 | 0.552 | 4.845 | 0.00 |

*Note: All models include subject as a random effect variable.*

**Table S1.** **Comparison of multiple models of time-lagged prediction of daily number of cigarettes smoked.**

${IV}_{fixed}$ indicates which variables were included as a fixed effect variable in the model. *Usage* variable is the number of cigarettes smoked on that day. *Craving, mood, stress, depression, anxiety* are self-report variables recorded on the day. *Medication* indicates if the participant took (1) the prescribed medication on that day or not (0). *⍺, - β,* and *log k* refer to the model parameters from the task completed on that day. *Day* indicates how many days it has been since the beginning of the clinic. *MSE=Mean Squared Errors.*

|  |  | Train | | | Test | | |
| --- | --- | --- | --- | --- | --- | --- | --- |
| # | ${IV}_{fixed}$ | $R^{2}$ | MSE | $\Delta MSE$ | $R^{2}$ | MSE | $\Delta MSE$ |
| 1 |  | 0.548 | 5.974 | 1.061 | 0.252 | 5.282 | 1.619 |
| 2 | 𝛼 | 0.551 | 5.932 | 1.019 | 0.243 | 5.349 | 1.686 |
| 3 | 𝛼, Day | 0.581 | 5.535 | 0.621 | 0.079 | 6.665 | 3.002 |
| 4 | −𝛽 | 0.549 | 5.962 | 1.049 | 0.249 | 5.315 | 1.653 |
| 5 | −𝛽, Day | 0.580 | 5.550 | 0.636 | 0.083 | 6.654 | 2.992 |
| 6 | log k | 0.554 | 5.898 | 0.984 | 0.241 | 5.357 | 1.694 |
| 7 | log k, Day | 0.581 | 5.534 | 0.620 | 0.084 | 6.624 | 2.962 |
| 8 | 𝛼, −𝛽, log k | 0.557 | 5.850 | 0.937 | 0.231 | 5.451 | 1.789 |
| 9 | 𝛼, −𝛽, log k, Day | 0.584 | 5.497 | 0.583 | 0.087 | 6.606 | 2.943 |
| 10 | Usage | 0.602 | 5.269 | 0.356 | 0.484 | 3.677 | 0.014 |
| 11 | Usage, Day | 0.613 | 5.130 | 0.217 | 0.446 | 3.939 | 0.276 |
| 12 | Usage, 𝛼 | 0.604 | 5.247 | 0.334 | 0.477 | 3.733 | 0.070 |
| 13 | Usage, 𝛼, Day | 0.614 | 5.112 | 0.199 | 0.441 | 3.983 | 0.320 |
| 14 | Usage, −𝛽 | 0.604 | 5.238 | 0.325 | 0.482 | 3.708 | 0.045 |
| 15 | Usage, −𝛽, Day | 0.614 | 5.103 | 0.190 | 0.449 | 3.935 | 0.272 |
| 16 | Usage, log k | 0.605 | 5.229 | 0.316 | 0.477 | 3.730 | 0.067 |
| 17 | Usage, log k, Day | 0.614 | 5.107 | 0.194 | 0.443 | 3.966 | 0.303 |
| 18 | Usage, Craving | 0.608 | 5.196 | 0.283 | 0.483 | 3.663 | 0.000 |
| 19 | Usage, Craving, Day | 0.616 | 5.084 | 0.171 | 0.451 | 3.884 | 0.221 |
| 20 | Usage, Craving, 𝛼, −𝛽, log k | 0.614 | 5.110 | 0.197 | 0.468 | 3.800 | 0.137 |
| 21 | Usage, Craving, 𝛼, −𝛽, log k, Day | 0.621 | 5.019 | 0.105 | 0.445 | 3.950 | 0.287 |
| 22 | Usage, Craving, Mood, Stress, Depression, Anxiety, Medication | 0.619 | 5.036 | 0.123 | 0.466 | 3.833 | 0.170 |
| 23 | Usage, Craving, Mood, Stress, Depression, Anxiety | 0.621 | 5.015 | 0.102 | 0.466 | 3.832 | 0.169 |
| 24 | Usage, Craving, Mood, Stress, Depression, Anxiety, Day | 0.622 | 5.004 | 0.091 | 0.446 | 3.937 | 0.275 |
| 25 | Usage, Craving, Mood, Stress, Depression, Anxiety, Medication, Day | 0.624 | 4.974 | 0.061 | 0.449 | 3.925 | 0.262 |
| 26 | Usage, Craving, Mood, Stress, Depression, Anxiety, 𝛼, −𝛽, log k | 0.615 | 5.097 | 0.184 | 0.479 | 3.714 | 0.051 |
| 27 | Usage, Craving, Mood, Stress, Depression, Anxiety, Medication, 𝛼, −𝛽, log k | 0.613 | 5.123 | 0.210 | 0.480 | 3.708 | 0.045 |
| 28 | Usage, Craving, Mood, Stress, Depression, Anxiety, 𝛼, −𝛽, log k, Day | 0.627 | 4.938 | 0.025 | 0.443 | 3.984 | 0.322 |
| 29 | Usage, Craving, Mood, Stress, Depression, Anxiety, Medication, 𝛼, −𝛽, log k, Day | 0.628 | 4.913 | 0.000 | 0.446 | 3.966 | 0.303 |

**Table S2.** **Comparison of multiple models of time-lagged prediction of daily number of cigarettes smoked, using time-aware cross validation.**

Model specifications and implementation details are identical to those presented in the **Table S1**. Under time-aware CV, models including *Day* as a fixed variable showed lower predictive performance as expected, because test folds necessarily contain day values larger than those observed during training, which creates a temporal extrapolation problem. We therefore focus on the following comparison of models without the *Day* variable.

Under time-aware CV, models showed slightly attenuated but broadly comparable predictive performance. For instance, the model including *Usage* and *Craving* achieved the best predictive performance among the list (Model 18: ${R^{2}}_{test}$ = 0.48, MSE = 3.66), and further inclusion of *Mood, Stress, Depression,* and *Anxiety* yielded comparable performance (Model 23: ${R^{2}}_{test}$ = 0.47, MSE = 3.83). Notably, models additionally incorporating computational task parameters performed marginally better (Model 26: ${R^{2}}_{test}$ = 0.48, Δ${R^{2}}_{test}$ ≈ 0.01 relative to Model 18, MSE = 3.71).

| **Predictor (Day_t_)** | | **Estimate** | **Standard error** | **t-value** | **p-value** | **ICC** | **RMSSD** |
| --- | --- | --- | --- | --- | --- | --- | --- |
| **# of observations** | | 2,072 |  |  |  |  |  |
| **Fixed effects** | | **Coefficient** |  |  |  |  |  |
| Intercept | | 2.16 | 0.19 | 11.61 | <0.001 |  |  |
| Task | alpha (⍺) | -0.07 | 0.08 | -0.93 | 0.351 | 0.75 | 0.36 |
|  | -beta (-β) | 0.27 | 0.09 | 2.93 | 0.003 | 0.75 | 0.46 |
|  | log(k) | 0.12 | 0.12 | 1.06 | 0.288 | 0.85 | 0.53 |
|  | Usage | 1.22 | 0.06 | 19.47 | <0.001 |  |  |
| Survey | Craving | 0.28 | 0.07 | 3.86 | <0.001 | 0.56 | 0.16 |
|  | Mood | 0.02 | 0.07 | 0.32 | 0.749 | 0.44 | 0.16 |
|  | Stress | -0.08 | 0.09 | -0.91 | 0.364 | 0.46 | 0.11 |
|  | Depression | 0.33 | 0.11 | 3.07 | 0.002 | 0.68 | 0.14 |
|  | Anxiety | 0.08 | 0.11 | 0.69 | 0.492 | 0.71 | 0.14 |
| Clinic-related variables | Medication | -0.37 | 0.13 | -2.93 | 0.003 |  |  |
|  | Day | -0.23 | 0.05 | -4.52 | <0.001 |  |  |
| **Random effects** | | **Variance** | **Standard deviation** |  |  |  |  |
| Subject (intercept) | | 2.70 | 1.64 |  |  |  |  |
| Residual | | 4.44 | 2.11 |  |  |  |  |

**Table S3.** **Time-lagged association of task and survey variables with prospective (next-day) smoking behavior.**

ICC=Intraclass correlation, RMSSD=Root mean square of successive differences.

| Index | Task | Self-report |  |  |
| --- | --- | --- | --- | --- |
| **A** | (Random)  One day  from week 1 | - | - Randomly sampled a single day within the first week  - Repeated sampling for 100 times |  |
|  |  |  |  |  |
| **B** | Mean week 1 | - | - Took all days within the first week and averaged them |  |
|  |  |  |  |  |
| **C** | Mean all weeks | - | - Took all days across all weeks and averaged them |  |
|  |  |  |  |  |
| **D** | Random  (Mean of 100 iters) | - | - Randomly sampled a single day from each week for all 5-6 week - 100 iterations of sampling |  |
|  |  |  |  |  |
| **E** | - | (Random)  One day  from week 1 | - Randomly sampled a single day within the first week - 100 iterations of sampling |  |
|  |  |  |  |  |
| **F** | - | Mean week 1 | - Took all days within the first week and averaged them |  |
|  |  |  |  |  |
| **G** | - | Mean all weeks | - Took all days across all weeks and averaged them |  |
|  |  |  |  |  |
| **H** | - | Random  (Mean of 100 iters) | - Randomly sampled a single day from each week for all 5-6 week - 100 iterations of sampling |  |
|  |  |  |  |  |
| **I** | (Random)  One day  from week 1 | (Random)  One day  from week 1 | - Randomly sampled a single day within the first week - 100 iterations of sampling |  |
|  |  |  |  |  |
| **J** | Mean week 1 | Mean week 1 | - Took all days within the first week and averaged them |  |
|  |  |  |  |  |
| **K** | Mean all weeks | Mean all weeks | - Took all days across all weeks and averaged them |  |
|  |  |  |  |  |
| **L** | Mean week 1 | Random  (Mean of 100 iters) | - Randomly sampled a single day from each week for all 5-6 week - 100 iterations of sampling |  |
|  |  |  |  |  |
| **M** | Random  (Mean of 100 iters) | Mean 1 week | - Task variables: Randomly sampled a single day from each week for all 5-6 weeks - Survey variables: Took all days within the first week and averaged them |  |
|  |  |  |  |  |
| **N** | Random  (Mean of 100 iters | Random  (Mean of 100 iters) | - Task variables: Took all days within the first week and averaged them  - Survey variables: Randomly sampled a single day from each week for all 5-6 weeks |  |
|  |  |  |  |  |

**Table S4.** **Description of each predictive model for smoking cessation with different inclusion criteria.** All the models include covariates of age, sex, and length of smoking history.

| Index | Task | Survey | Alpha | 0 | 0.1 | 0.2 | 0.3 | 0.4 | 0.5 | 0.6 | 0.7 | 0.8 | 0.9 | 1.0 |
| --- | --- | --- | --- | --- | --- | --- | --- | --- | --- | --- | --- | --- | --- | --- |
|  |  |  | Set | 10 folds | | | | | | | | | | |
| A | One day from week 1 | - | Train | 0.82 | 0.82 | 0.82 | 0.82 | 0.82 | 0.81 | 0.81 | 0.81 | 0.82 | 0.81 | 0.81 |
|  |  |  | Test | 0.71 | 0.71 | 0.71 | 0.71 | 0.72 | 0.71 | 0.71 | 0.71 | 0.72 | 0.71 | 0.71 |
| B | Mean  week 1 | - | Train | 0.81 | 0.81 | 0.81 | 0.80 | 0.80 | 0.81 | 0.80 | 0.81 | 0.80 | 0.81 | 0.80 |
|  |  |  | Test | 0.72 | 0.73 | 0.73 | 0.74 | 0.74 | 0.74 | 0.74 | 0.74 | 0.74 | 0.73 | 0.74 |
| C | Mean  all weeks | - | Train | 0.81 | 0.81 | 0.81 | 0.80 | 0.80 | 0.81 | 0.80 | 0.81 | 0.80 | 0.80 | 0.80 |
|  |  |  | Test | 0.71 | 0.72 | 0.73 | 0.73 | 0.74 | 0.73 | 0.74 | 0.74 | 0.74 | 0.74 | 0.74 |
| D | Random  all weeks | - | Train | 0.82 | 0.82 | 0.82 | 0.82 | 0.82 | 0.82 | 0.82 | 0.82 | 0.82 | 0.81 | 0.81 |
|  |  |  | Test | 0.71 | 0.72 | 0.72 | 0.73 | 0.73 | 0.73 | 0.73 | 0.73 | 0.73 | 0.73 | 0.73 |
| E | - | One day from week 1 | Train | 0.82 | 0.82 | 0.82 | 0.82 | 0.82 | 0.81 | 0.81 | 0.81 | 0.82 | 0.81 | 0.81 |
|  |  |  | Test | 0.71 | 0.71 | 0.71 | 0.71 | 0.71 | 0.71 | 0.72 | 0.72 | 0.71 | 0.71 | 0.72 |
| F | - | Mean  week 1 | Train | 0.81 | 0.81 | 0.81 | 0.80 | 0.81 | 0.80 | 0.80 | 0.80 | 0.80 | 0.81 | 0.80 |
|  |  |  | Test | 0.71 | 0.73 | 0.72 | 0.73 | 0.72 | 0.73 | 0.73 | 0.73 | 0.73 | 0.72 | 0.72 |
| G | - | Mean  all weeks | Train | 0.84 | 0.84 | 0.84 | 0.84 | 0.84 | 0.84 | 0.84 | 0.84 | 0.84 | 0.84 | 0.83 |
|  |  |  | Test | 0.76 | 0.77 | 0.78 | 0.78 | 0.79 | 0.79 | 0.79 | 0.79 | 0.79 | 0.79 | 0.79 |
| H | - | Random  all weeks | Train | 0.87 | 0.87 | 0.87 | 0.87 | 0.87 | 0.86 | 0.86 | 0.86 | 0.86 | 0.86 | 0.86 |
|  |  |  | Test | 0.78 | 0.79 | 0.79 | 0.79 | 0.79 | 0.79 | 0.80 | 0.80 | 0.80 | 0.80 | 0.80 |
| I | One day from week 1 | One day from week 1 | Train | 0.86 | 0.86 | 0.86 | 0.86 | 0.85 | 0.85 | 0.85 | 0.85 | 0.85 | 0.84 | 0.85 |
|  |  |  | Test | 0.73 | 0.73 | 0.74 | 0.73 | 0.73 | 0.73 | 0.73 | 0.73 | 0.73 | 0.72 | 0.73 |
| J | Mean  week 1 | Mean  week 1 | Train | 0.86 | 0.86 | 0.86 | 0.86 | 0.86 | 0.86 | 0.86 | 0.86 | 0.86 | 0.86 | 0.85 |
|  |  |  | Test | 0.74 | 0.75 | 0.76 | 0.77 | 0.76 | 0.76 | 0.76 | 0.76 | 0.76 | 0.76 | 0.75 |
| K | Mean  all weeks | Mean  all weeks | Train | 0.90 | 0.89 | 0.89 | 0.90 | 0.89 | 0.89 | 0.89 | 0.89 | 0.88 | 0.89 | 0.89 |
|  |  |  | Test | 0.79 | 0.80 | 0.81 | 0.82 | 0.82 | 0.82 | 0.83 | 0.83 | 0.83 | 0.83 | 0.83 |
| L | Mean  week 1 | Random (Mean of 100 iterations) | Train | 0.90 | 0.90 | 0.90 | 0.90 | 0.90 | 0.90 | 0.90 | 0.90 | 0.90 | 0.90 | 0.90 |
|  |  |  | Test | 0.80 | 0.81 | 0.82 | 0.82 | 0.82 | 0.82 | 0.82 | 0.82 | 0.82 | 0.82 | 0.82 |
| M | Random (Mean of 100 iterations) | Mean  week 1 | Train | 0.87 | 0.87 | 0.87 | 0.87 | 0.87 | 0.87 | 0.87 | 0.87 | 0.86 | 0.86 | 0.86 |
|  |  |  | Test | 0.75 | 0.76 | 0.76 | 0.76 | 0.76 | 0.76 | 0.76 | 0.76 | 0.76 | 0.76 | 0.76 |
| N | Random (Mean of 100 iterations) | Random (Mean of 100 iterations) | Train | 0.90 | 0.90 | 0.90 | 0.90 | 0.90 | 0.90 | 0.89 | 0.89 | 0.89 | 0.89 | 0.89 |
|  |  |  | Test | 0.79 | 0.80 | 0.81 | 0.81 | 0.82 | 0.82 | 0.82 | 0.82 | 0.82 | 0.82 | 0.82 |

**Table S5**. **Comparison of model performances across different combinations of task and self-report variables.**

Here, *alpha* indicates the penalty term for elastic net regression. Alpha value of 0.6 showed the best in all of the models, thus reported in the main text. Each value indicates the mean area under curve (AUC) score after 1,000 iterations. We show four different types of data preprocessing for each modality (task & survey). *One day from week 1* indicates that a datapoint within the first week was randomly pulled, *mean week 1* indicates that the mean value of the datapoints within the first week were used, *mean all weeks* indicates that the mean value of all the datapoints throughout the data collection (5-6 weeks) was used, and the *random all weeks* indicates that one value from each and every week was randomly pulled, to be averaged (across 100 iterations) and used for the prediction.

| Task | % Converged | Mean refits | Median refits | Max refits | % 1st fit |
| --- | --- | --- | --- | --- | --- |
| **CRA** | 99.8 | 1.7 | 1 | 100 | 88.7 |
| **DDT** | 99.0 | 2.2 | 1 | 100 | 94.8 |

**Table S6. Summary of convergence and refitting behavior for computational model estimation.**
